# The N-Terminal β-Sheet of Peroxiredoxin 4 in the Large Yellow Croaker *Pseudosciaena crocea* Is Involved in Its Biological Functions

**DOI:** 10.1371/journal.pone.0057061

**Published:** 2013-02-25

**Authors:** Yinnan Mu, Fu-Ming Lian, Yan-Bin Teng, Jingqun Ao, Yong-Liang Jiang, Yong-Xing He, Yuxing Chen, Cong-Zhao Zhou, Xinhua Chen

**Affiliations:** 1 Key Laboratory of Marine Biogenetic Resources, Third Institute of Oceanography, State Oceanic Administration, Xiamen, Fujian, People’s Republic of China; 2 Hefei National Laboratory for Physical Sciences at Microscale and School of Life Sciences, University of Science and Technology of China, Hefei, Anhui, People’s Republic of China; Instituto de Biociencias - Universidade de São Paulo, Brazil

## Abstract

Peroxiredoxins (Prxs) are thiol-specific antioxidant proteins that exhibit peroxidase and peroxynitrite reductase activities involved in the reduction of reactive oxygen species. The peroxiredoxin Prx4 from the large yellow croaker *Pseudosciaena crocea* is a typical 2-Cys Prx with an N-terminal signal peptide. We solved the crystal structure of Prx4 at 1.90 Å and revealed an N-terminal antiparallel β-sheet that contributes to the dimer interface. Deletion of this β-sheet decreased the *in vitro* peroxidase activity to about 50% of the wild-type. *In vivo* assays further demonstrated that removal of this β-sheet led to some impairment in the ability of Prx4 to negatively regulate nuclear factor-*κ*B (NF-*κ*B) activity and to perform its role in anti-bacterial immunity. These results provide new insights into the structure and function relationship of a peroxiredoxin from bony fish.

## Introduction

Peroxiredoxins (Prxs) are thiol-specific antioxidant proteins that protect cells against reactive oxygen species (ROS) such as hydrogen peroxide, alkyl peroxides and peroxynitrite, through their peroxidase and peroxynitrite reductase activities [1], [2], [3], [4]. Prxs reduce peroxides with the redox-active cysteines, which distinguish them from other peroxidases that require cofactors such as metal ions or prosthetic groups [5]. Prxs have been identified in almost all organisms, normally existing in several isoforms and expressed at a relatively high level [4], [6]. Based on distinct positions of the reactive Cys residues, Prxs have been classified into three groups: typical 2-Cys, atypical 2-Cys and 1-Cys Prxs [4]. Prxs in all subfamilies, however, possess one strictly conserved cysteine called peroxidatic cysteine (C_P_), which is located in the N-terminal region. The reaction could be dissected into three steps. First, the sulfur atom of C_P_ residue executes a nucleophilic attack at the O-O bond of the peroxide substrate at the cost of being oxidized into the cysteine sulfenic acid form. The second step differs in three subfamilies of Prx. For Prxs in the typical 2-Cys subfamily, dimerization is necessary for the activity. The second redox-active cysteine called resolving cysteine (C_R_) located in the C-terminal of another subunit attacks the cysteine sulfenic acid resulting in formation of an intermolecular disulfide bond. In contrast, for the atypical 2-Cys Prxs, the C_R_ residue is located within the same subunit and executes the nucleophilic attack to form an intramolecular disulfide bond. This step does not occur with the 1-Cys Prxs, due to the lack of the C_R_ residue. Finally, the oxidized Prx is regenerated by thiol-containing electron donors such as thioredoxin, glutathione, or DL-Dithiothreitol (DTT) [4], [5], [7]. For typical eukaryotic 2-Cys Prxs, the cysteine sulfenic acid reacts with a second peroxide molecule to form a cysteine sulfinic acid, which can be reversed by the sulfinic acid reductase sulfiredoxin in an ATP-dependent manner [8], [9], [10]. The activity of Prx is irreversibly lost once the cysteine sulfinic acid is further oxidized to cysteine sulfonic acid [4]. In addition to the antioxidant activity, Prxs are also involved in cell proliferation, differentiation, gene expression and intracellular signaling pathways [11], [12], [13].

Since the first crystal structure of Prx was determined in 1998 [14], a series of structures of Prxs at various redox and/or hydrogen peroxide-binding states have been reported [15], [16], [17]. All of these structures share a conserved thioredoxin fold. In all reduced Prxs, the C_P_ residue is located in the first turn of a helix and surrounded by three highly conserved residues (Pro, Thr and Arg). Upon oxidation, the residues adjacent to C_P_ and C_R_ undergo significant conformational changes to form the disulfide bond in the oxidized form [15]. Moreover, the typical 2-Cys Prxs can assemble into the toroid-shaped homodecameric complexes, which are related to the redox states and/or functions [5], [18].

Human Prx4 is a typical 2-Cys Prx with an N-terminal signal peptide [19], [20]. In addition to the peroxidase activity, it also plays a role in inhibiting NF-*κ*B function as a cytosolic molecule [21], or activating NF-*κ*B as an extracellular factor [22]. Recently, we identified the gene of Prx4 from the large yellow croaker *Pseudosciaena crocea*, and found that it regulated the pro-inflammatory responses by inhibiting NF-*κ*B activity and protected the fish against bacterial challenge [23]. Here, we report the 1.90 Å crystal structure of *P. crocea* Prx4 in the reduced form. Comparative structural analysis revealed an N-terminal antiparallel β-sheet, which is located at the dimeric interface and involved in the *in vitro* enzymatic activity and *in vivo* biological functions of *P. crocea* Prx4.

## Materials and Methods

### Ethics Statement

This study was carried out in strict accordance with the Regulations for the Administration of Affairs Concerning Experimental Animals established by the Fujian Provincial Department of Science and Technology. The experiments on this animal were approved by the Animal Care and Use Committee of the Third Institute of Oceanography, State Oceanic Administration. All surgery was performed under Tricaine-S anesthesia, and all efforts were made to minimize suffering.

### Cloning, Expression and Purification of *P. crocea* Prx4

The DNA sequence encoding the mature Prx4 protein without the 29-residue signal peptide, as predicted by SignalP 3.0 Server [24] was amplified by PCR using the recombinant plasmid Prx4-pET28a (Novagen, Germany) as the template, which was constructed as previously described [23]. The PCR product was cloned into a pET28a-derived vector. This construct contained a hexahistidine (6× His) tag at the N-terminus of the recombinant protein, which was overexpressed in *E. coli* Rosetta (DE3) strain (Novagen, Germany) in 2× YT medium (Oxoid Ltd, England) supplemented with 34 µg/ml kanamycin and 30 µg/ml chloramphenicol. The cells were grown at 37°C to an A_600 nm_ of 0.6 and induced with 0.2 mM isopropyl-β-D-thiogalactoside for 4 hr at 37°C. Cells were harvested by centrifugation at 4000 g for 10 min and resuspended in lysis buffer (20 mM Tris-HCl, pH 8.5, 200 mM NaCl). After three cycles of freezing-thawing followed by sonication, the disrupted cells were centrifuged at 16,000 g for 25 min. The supernatant containing the target protein was collected and loaded onto a Ni-NTA column (Qiagen, Germany) equilibrated with the binding buffer (20 mM Tris-HCl, pH 8.5, 200 mM NaCl, 14 mM β-mercaptoethanol). The target protein was eluted with 500 mM imidazole buffer and further purified by gel filtration in a Superdex™ 200 column (GE Healthcare, USA) equilibrated with the binding buffer. Fractions containing the target protein were collected and concentrated to 15 mg/mL. The purity of the protein was evaluated by sodium dodecyl sulfate polyacrylamide gel electrophoresis and the protein sample was stored at −80°C until use. The Prx4C113S and truncated versions of Prx4ΔN62 and Prx4ΔN67, that lacked the N-terminal 62 and 67 residues respectively, were prepared with a similar procedure.

### Crystallization, Data Collection and Processing

The crystals were grown at 16°C using the hanging drop vapor-diffusion techniques. In each drop, 1 µL of the Prx4 at 15 mg/mL in the buffer of 20 mM Tris-HCl, pH 8.5, 50 mM NaCl, 14 mM β-mercaptoethanol, 10 mM DTT was mixed with 1 µL reservoir solution (10% polyethylene glycol monomethyl ether 5,000, 0.1 M HEPES-NaOH, pH 8.0) and equilibrated against 0.5 mL reservoir solution. Typically, crystals appeared in one day and reached the maximum size of 100×100×400 µm^3^ in one week. A single crystal was transferred to the cryoprotectant, which consisted of the reservoir solution plus 30% glycerol, and flash-frozen in liquid nitrogen. The X-ray diffraction data were collected at a wavelength of 0.9999 Å at the Shanghai Synchrotron Radiation Facility (SSRF) using beamline 17 U with a MX225 CCD (MARresearch, Germany). The diffraction data were indexed, integrated, and scaled with HKL2000 [25].

### Structure Solution and Refinement

The crystal structure of Prx4 was determined by molecular replacement with the program *MOLREP* [26] using the coordinates of human truncated Prx4 (PDB code 2PN8) as the search model. The initial model was refined using the maximum likelihood method implemented in *REFMAC5* [27] as part of the *CCP4i* [28] program suite and rebuilt interactively using the σ_A_-weighted electron density maps with coefficients 2mFo-DFc and mFo-DFc in the program *COOT* [29]. Five percent of the reflections were set aside to calculate an R-free factor. Refinement finally converged to an R-factor of 19.56% and an R-free factor of 21.98% at 1.90 ? resolution. The final model was validated with the programs *MOLPROBITY* [30] and *PROCHECK* [31]. The statistics and refinement parameters are listed in Table 1. All structure figures were prepared with the program *PyMol* [32]. The final structure factors and coordinates of *P. crocea* Prx4 have been deposited in the Protein Data Bank under the accession code of 3QPM.

**Table 1:** Crystal parameters, data collection and structure refinement statistics.

*Data processing*	
Space group	*C2*
Unit cell (Å), (°)	a = 145.12, b = 196.00, c = 51.74, α = γ = 90.00, β = 105.23
Resolution range (Å)	50.00–1.90 (1.97–1.90)^a^
Unique reflections	108,135 (10,312)
Completeness (%)	98.9 (94.2)
* <I/σ(I)>*	12.4 (5.2)
R_merge_ b (%)	6.6 (21.4)
Redundancy	3.5
*Refinement statistics*	
Resolution range (Å)	37.98–1.90
R-factor^c^/R-free^d^ (%)	19.56/21.98
Number of protein atoms	7,784
Number of water atoms	624
RMSD^e^ bond lengths (Å)	0.014
RMSD bond angles (°)	1.281
Mean B factors (Å^2^)	33.51
*Ramachandran plot* f *(residues, %)*	
Most favored (%)	97.41
Additional allowed (%)	2.59
Outliers (%)	0
PDB entry	3QPM

a The values in parentheses refer to statistics in the highest bin.

b R_merge_ = ∑_hkl_∑_i_|I_i_(hkl)- <I(hkl)>|/∑_hkl_∑_i_I_i_(hkl), where I_i_(hkl) is the intensity of an observation and <I(hkl)> is the mean value for its unique reflection; Summations are over all reflections.

c R-factor = ∑_h_|Fo(h)-Fc(h)|/∑_h_Fo(h), where Fo and Fc are the observed and calculated structure-factor amplitudes, respectively.

d R-free was calculated with 5% of the data excluded from the refinement.

e Root-mean square-deviation from ideal values.

f Categories were defined by Molprobity.

### Enzymatic Assays

Peroxidase activity was determined by a spectrophotometric assay that monitored the consumption of NADPH at 340 nm, as described previously [33]. The *Saccharomyces cerevisiae* thioredoxin Trx1 and thioredoxin reductase Trr1 were prepared as previously described [34], [35] in buffer containing 20 mM Tris-HCl, pH 7.0 and 100 mM NaCl. The reaction was carried out in a final volume of 200 µL reaction mix containing 50 mM HEPES-NaOH, pH 7.0, 250 µM NADPH, 3 µM Trx1, 1.5 µM Trr1, 1 µM purified Prx4 (Prx4ΔN62, Prx4ΔN67 or Prx4C113S), and 50 µM H_2_O_2_. Following the reaction triggered by the addition of H_2_O_2_, the consumption of NADPH was monitored for 3 min, with a step of 15 sec at room temperature with a DU800 spectrophotometer (Beckman Coulter, Fullerton, CA, USA). All assays were repeated three times.

### Fish Culture, Protein Injection, and Sample Preparation

Large yellow croakers with an average weight of 100 g were purchased from a mariculture farm in Lianjiang, Fujian, China. After acclimatization in an aerated seawater tank for three days, four groups of 30 fish each were injected intramuscularly with purified Prx4, Prx4ΔN62, Prx4ΔN67 and Prx4C113S protein (100 µg/100 g fish) or sterile 0.85% NaCl (100 µL/100 g fish), respectively. The 0.85% NaCl-injected group served as a negative control. Spleen tissues were collected from six fish in each group at different times after injection (0, 12, 24, 48 and 72 hr) and then flash-frozen in liquid nitrogen. Three pooled samples from each treatment were subjected to electrophoretic mobility shift assay (EMSA). All experiments were repeated three times.

### Electrophoretic Mobility Shift Assay (EMSA) and Bacterial Challenge Experiments

EMSA was performed as described previously [23]. In the bacterial challenge experiments, five groups of 50 large yellow croakers each were injected intramuscularly as follows: (1) Prx4 protein (100 µg/100 g fish), (2) Prx4ΔN62 protein (100 µg/100 g fish), (3) Prx4ΔN67 protein (100 µg/100 g fish), (4) Prx4C113S protein (100 µg/100 g fish) and (5) 0.85% NaCl (100 µL/100 g fish). Twelve hours after injection, fish were infected with mixed bacteria (5×10^6^ cfu/mL of *Vibrio alginolyticus*, *Vibrio parahaemolyticus,* and *Aeromonas hydrophila*) at a dose of 200 µL/100 g fish. Mortality was monitored for 5 days after injection.

## Results

### The Overall Structure and Active Site

**Figure 1:**
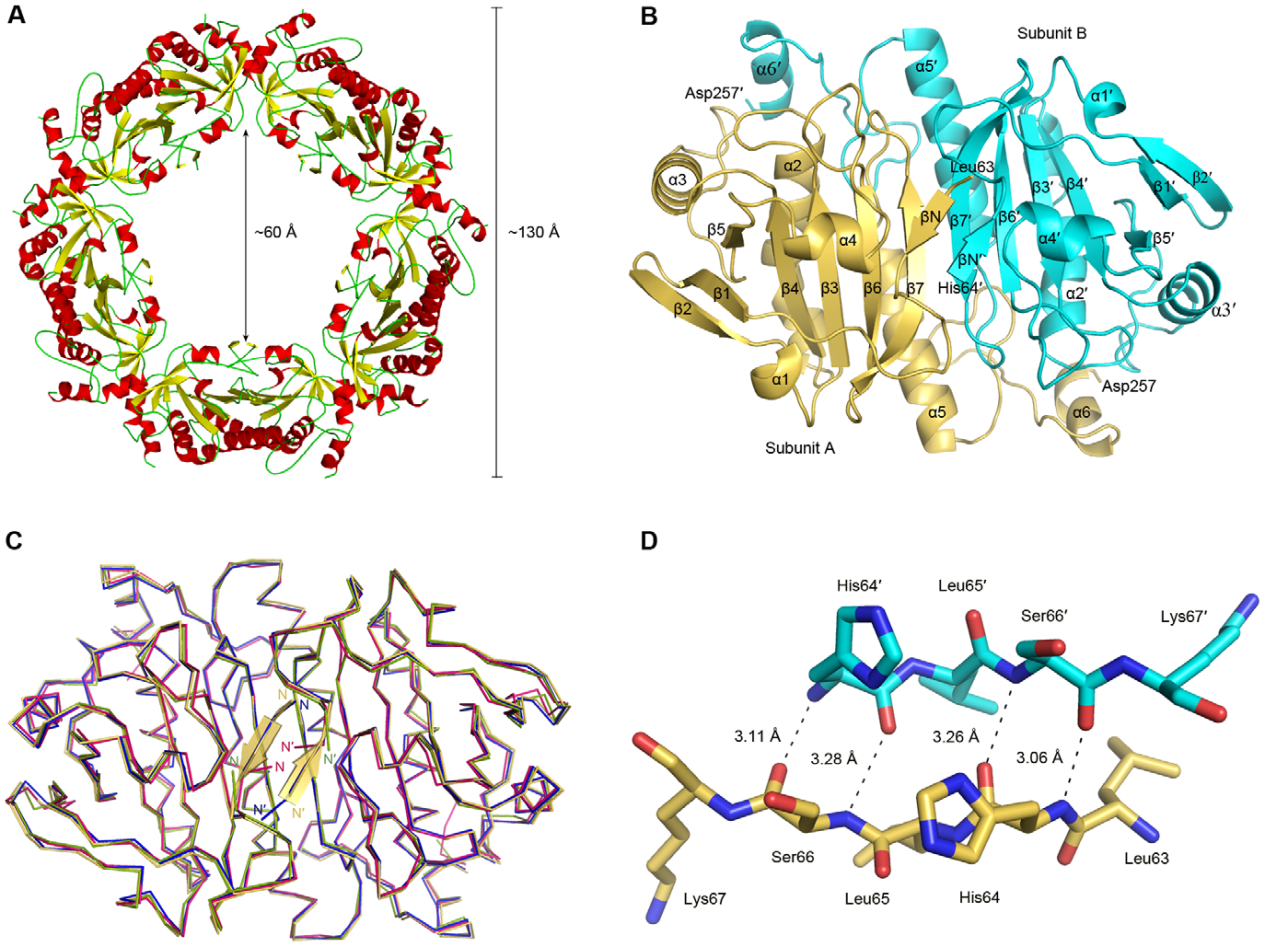
Structure and comparison of *P. crocea* Prx4. **A** The homodecameric structure. **B** The dimeric structure. Subunits A and B are colored in orange and cyan, respectively. All secondary structures are labeled sequentially. **C** Superposition of *P. crocea* Prx4 (orange), *H. sapiens* Prx4 (blue), *R. norvegicus* Prx1 (hotpink), and *T. cruzi* TryP (green). **D** Hydrogen bonds of the antiparallel β-sheet. Atoms N and O are colored in blue and red, respectively, in both subunits. Atom C is colored in orange and cyan in subunit A and B, respectively.

The *P. crocea* Prx4 also assembles into a toroid-shaped homodecameric complex as other typical 2-Cys Prxs [5], both in the crystal structure and in the solution as indicated by gel filtration (data not shown). Each asymmetric unit contains five subunits, two pairs of dimers and a monomer. Symmetric processing enables us to define a decamer (pentamer of dimers), with an inner diameter of ∼60 Å and an outer diameter of ∼130 Å (**Fig. 1A**). The basic unit of functional *P. crocea* Prx4 is a homodimer (**Fig. 1B**). The final structural model of this basic functional unit spans 389 residues (Leu63–Asp257 in subunit A, and His64′–Asp257′ in subunit B). The 33 N-terminal residues (Glu30–Ser62) and the three C-terminal residues (Lys258–Lys260) are not fitted into the final model due to the weak electron density, implying a high degree of structural flexibility of these regions. The core structure adopts a typical thioredoxin fold, which is composed of five β-strands (β3, β4, β5, β6 and β7) and four α-helices (α2, α3, α4 and α5). The C-terminal protrusion (Gly231–Asp257), which was comprised of helix α6 and a long loop succeeding helix α5, interacts with the core domain (Leu63–His230) of the other subunit and contributes considerably to the dimeric interface. In addition, the pair of N-terminal βN strands forms a short antiparallel β-sheet, packing against the β-sheet of β7–β7′.

Similar to previously determined Prx structures, the C_P_ residue Cys113 is located in the first turn of helix α2 and is surrounded by three highly conserved residues Pro106, Thr110 and Arg189. The distance between the sulphur atoms of C_P_ (Cys113) and C_R_ (Cys234′) in the other subunit of the homodimer is approximately 14 Å. Thus, as proposed previously [15], large conformational changes are required in order for *P. crocea* Prx4 to form the intermolecular disulfide bond between C_P_ and C_R_.

### Structural Comparison of *P. crocea* Prx4 and Other Typical 2-Cys Prxs

Superposition of the *P. crocea* Prx4 dimer on the human Prx4 (PDB code, 3TJK) [36], *Rattus norvegicus* Prx1 (2Z9S) [37], and *Trypanosoma cruzi* TryP (1UUL) [38] yields overall root mean square deviations of 0.41 Å, 0.59 Å and 0.60 Å over 389, 378 and 378 Cα atoms, respectively. These 2-Cys Prxs share a similar overall structure (**Fig. 1C**). The most significant difference is within the N-terminal region. Similar to human Prx4 (3TJK), *P. crocea* Prx4 also possesses an antiparallel β-sheet located at the dimer interface (**Fig. 1C**). This β-sheet is stabilized via four main-chain hydrogen bonds (**Fig. 1D**).

### The Deletion of N-terminal β-sheet Affected the Peroxidase Activity of Prx4

**Figure 2:**
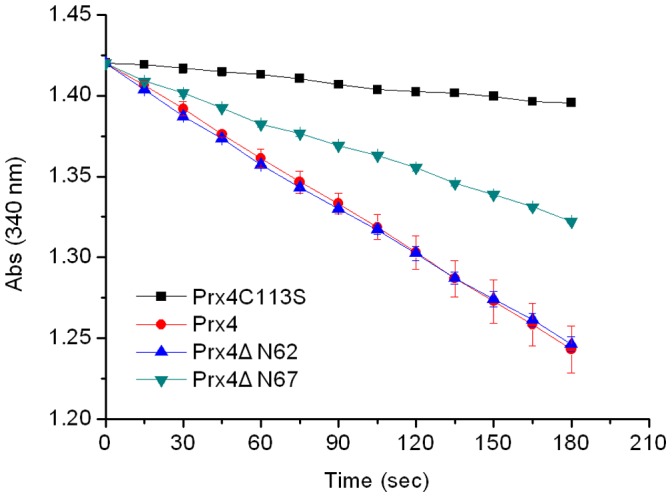
Peroxidase activity assays. Assays were performed with Prx4 (red circles), Prx4ΔN62 (blue triangles), Prx4ΔN67 (cyan inverted triangles) and Prx4C113S (black squares).

To determine whether the residues of the N-terminal β-strand (Leu63–Lys67) are necessary for the peroxidase activity of Prx4, we constructed two truncated versions of Prx4, referred to as Prx4ΔN62 (residues Leu63–Lys260) and Prx4ΔN67 (residues Ala68–Lys260). Using thioredoxin system (Trx1-Trr1-NADPH) as the electron donor, the reaction velocity of Prx4ΔN62 is about two times that of Prx4ΔN67, and is almost the same as that of Prx4 (**Fig. 2**). As could be expected, the C113S mutant abolishes the peroxidase activity. These results indicated that the N-terminal β-strand (residues Leu63–Lys67) contributed to the enzymatic activity of *P. crocea* Prx4.

### Importance of the N-terminal β-sheet for the Negative Regulation of NF-*κ*B Activity

**Figure 3:**
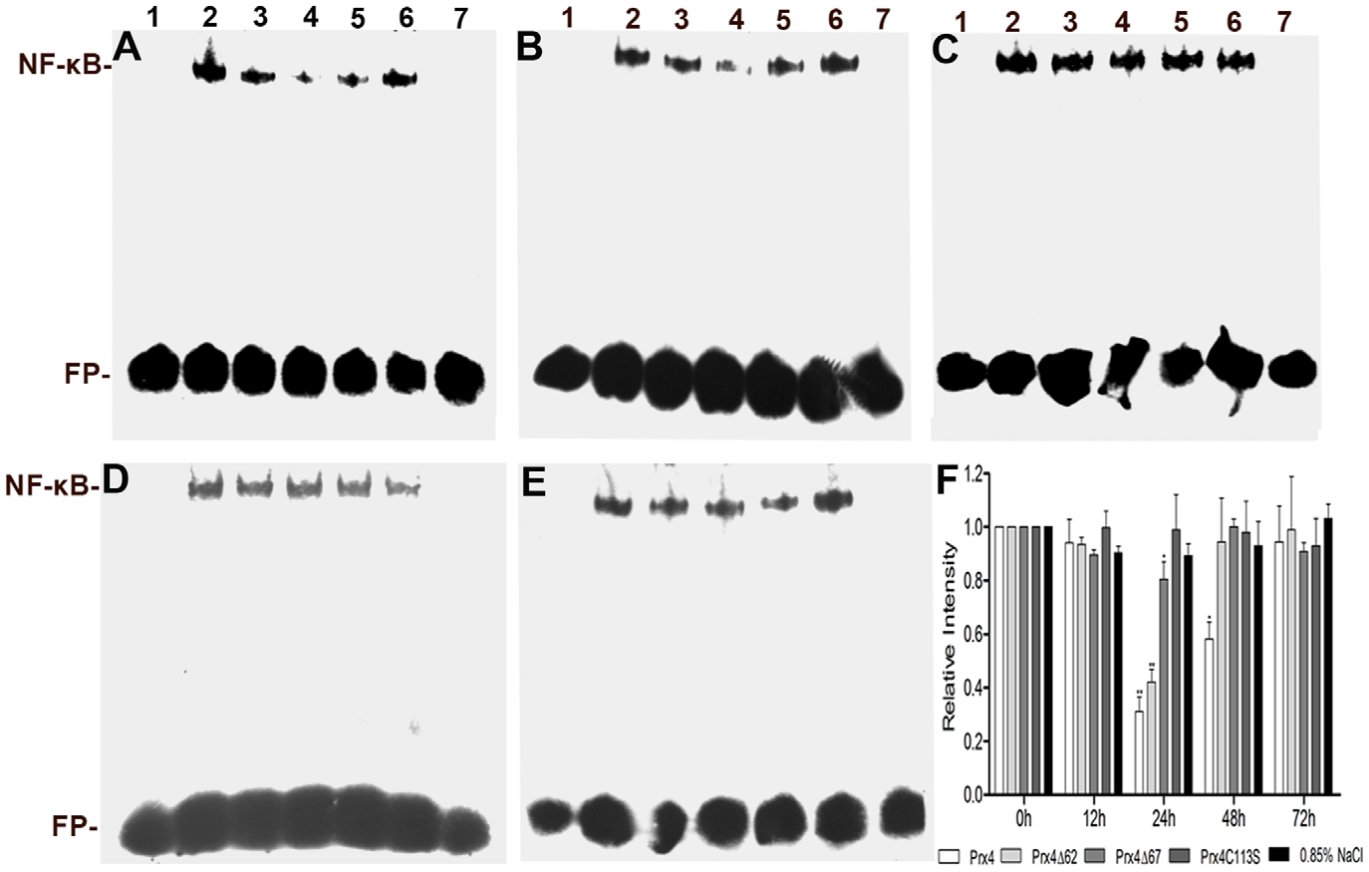
Effects of Prx4, Prx4ΔN62, Prx4ΔN67 and Prx4C113S protein on NF-*κ*B activation. EMSA of NF-*κ*B activity in nuclear extracts of spleen tissues from large yellow croakers injected with **A** Prx4, **B** Prx4ΔN62 protein, **C** Prx4ΔN67 protein, **D** Prx4C113S protein and **E** 0.85% NaCl (as a control). Lane 1 contains probe incubated without nuclear extracts; Lanes 2–6 contain nuclear extracts of spleen tissues collected at 0, 12, 24, 48, and 72 h post-injection; Lane 7 contains 100-fold excess of unlabeled oligonucleotide used to compete for binding. **F** Quantitation of relative activity of NF-*κ*B was performed with a TANON GIS Image System Ver.3.73.

Previous studies have showed that *P. crocea* Prx4 negatively regulates NF-*κ*B activity, thus modulating pro-inflammatory responses [23]. To determine whether the missing N-terminal β-sheet affects the function of *P. crocea* Prx4, Prx4ΔN62, Prx4ΔN67 and Prx4C113S were injected into *P. crocea*, and EMSA was performed to detect the NF-*κ*B activity in spleen tissues of fish from each treatment group. In Prx4ΔN62-injected fish, the NF-*κ*B activity was significantly decreased (−2.5-fold) at 24 hr after administration, similar to that in Prx4-injected fish (**Fig. 3A**), indicating that Prx4ΔN62 negatively regulated activation of NF-*κ*B. In Prx4ΔN67-injected fish, however, NF-*κ*B activity in the spleens remained virtually a little down-regulated (−1.25-fold) at 24 hr, similar to that in the Prx4C113S-injected fish (−1.11-fold) and 0.85% NaCl-injected fish (negative control), indicating that Prx4ΔN67, missing the N-terminal β-sheet, has a decreased ability to downregulate NF-*κ*B activity (**Fig. 3B, C and D**). These results suggest the involvement of the N-terminal β-sheet of *P. crocea* Prx4 in the negative regulation of NF-κB activity.

### Deletion of the N-terminal β-sheet Affects the Role of *P. crocea* Prx4 in the Anti-bacterial Immunity

**Figure 4:**
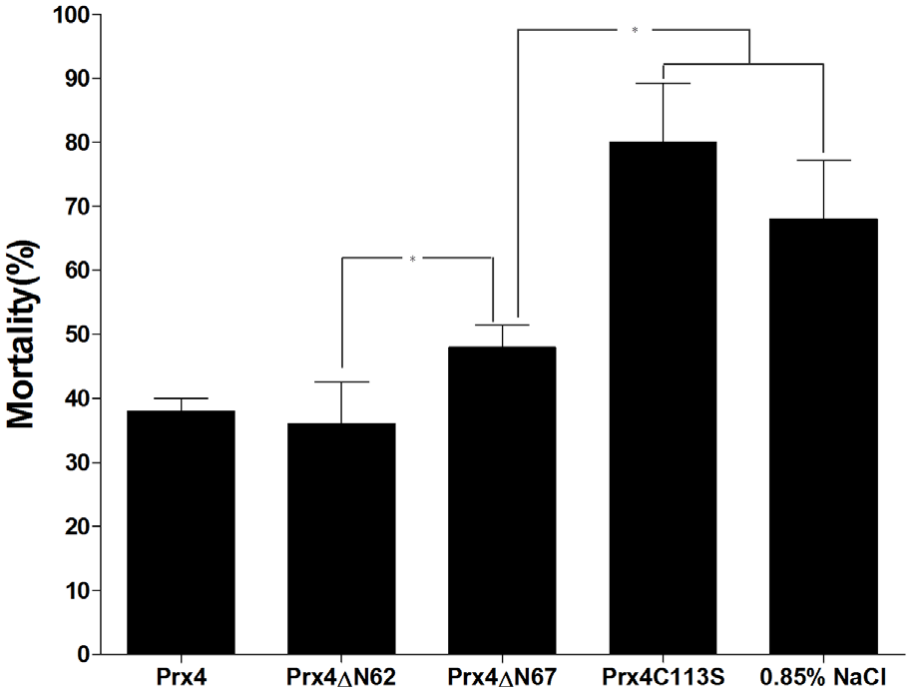
The functions of the three Prx4 proteins during bacterial challenge. Large yellow croakers were injected with Prx4, Prx4ΔN62, Prx4ΔN67, Prx4C113S or 0.85% NaCl and then challenged with a bacterial mixture. Fish mortality in each group was monitored for 5 days. The symbol * represents a significant difference in the mortality rate between the two groups injected with different proteins as indicated (*P*<0.05).

To further assess whether a lack of the N-terminal β-sheet affects the role of Prx4 in the immune response against bacterial challenge, four groups of healthy large yellow croakers were injected intramuscularly with Prx4, Prx4ΔN62, Prx4ΔN67, Prx4C113S, or 0.85% NaCl, followed by infection with the bacterial mixture. Five days after challenge, the mortality rate for the Prx4ΔN67-injected group was 48%, which was significantly higher than that of the Prx4ΔN62 and Prx4-injected group (36% and 38% mortality rate, *P*<0.05) (**Fig. 4**). The mortality rate of Prx4C113S - and 0.85% NaCl-injected groups was 68% and 80% (**Fig. 4**). These results indicated that Prx4ΔN62 possessed a higher protective effect against bacterial challenge compared to Prx4ΔN67, although Prx4ΔN67 also had a protective effect, suggesting that deletion of the N-terminal β-sheet may affect the role of this enzyme in anti-bacterial immunity in large yellow croakers.

## Discussion

In the present study, we determined the crystal structure of Prx4 from large yellow croaker, a marine fish species, for the first time. Similar to previously determined typical 2-Cys Prx structures [5], [15], *P. crocea* Prx4 also assembles into the toroid-shaped homodecameric complex with generally identical inner diameter and outer diameter (**Fig. 1A**). The B-type interface [15] is also adopted in *P. crocea* Prx4 homodimer (**Fig. 1B**). Structural superposition of *P. crocea* Prx4 dimer against other typical 2-Cys Prxs indicates that they share a similar overall structure. However, an extra N-terminal antiparallel β-sheet is found at the dimer interface of Prx4 proteins from *P. crocea* as well as *H. sapiens* (**Fig. 1C**). Since the basic functional unit of *P. crocea* Prx4 is a homodimer, this N-terminal β-sheet, which is situated at the dimer interface and involved in four main-chain hydrogen bonds (**Fig. 1D**), should be important for the peroxidase activity and biological function of this enzyme.

Several lines of evidence indicate that NF-*κ*B activation can be controlled by ROS, such as H_2_O_2_ and superoxide [21], [39]. Prxs govern intracellular H_2_O_2_ levels via their peroxidase activity, thus regulating NF-*κ*B activity [11], [21], [40]. Therefore, the peroxidase activity of Prxs within cells is essential for regulation of NF-*κ*B activity. Here, we demonstrated that Prx4ΔN67, which lacks the N-terminal β-sheet, exhibits a decreased peroxidase activity compared with Prx4ΔN62 (**Fig. 2**) and also has a decreased ability to downregulate NF-*κ*B activity (**Fig. 3B and C**). These results indicate that deletion of the N-terminal β-sheet decreased the activity of *P. crocea* Prx4, which may result in influence of its function in the negative regulation of NF-*κ*B activation due to the change of redox status within cells. Therefore, the N-terminal β-sheet may be involved in Prx4 regulation of NF-*κ*B activation by affecting the peroxidase activity of this enzyme.

NF-*κ*B is clearly one of the most important regulators of pro-inflammatory response, as synthesis of pro-inflammatory cytokines, such as TNF-α, IL-1β, and chemokines, is mediated by NF-*κ*B [41]. Studies have shown that *P. crocea* Prx4 may regulate pro-inflammatory responses by inhibiting NF-*κ*B activity and protected fish against bacterial challenge [23]. In this study, Prx4ΔN67 missing the N-terminal β-sheet has a decreased ability to downregulate NF-*κ*B activity, thus having lower potential to modulate pro-inflammatory response as compared with Prx4ΔN62. This may result in a higher mortality rate for the Prx4ΔN67-injected fish (48% mortality rate) than that for Prx4ΔN62-injected fish (36% mortality rate) in bacterial challenge experiments (**Fig. 4**).

In summary, we determined the 1.90 Å crystal structure of *P. crocea* Prx4 and identified an antiparallel β-sheet at the N-terminus. This β-sheet, which contributes to the dimer interface, is involved not only in the peroxidase activity of *P. crocea* Prx4 but also in the negative regulation of NF-*κ*B activity and anti-bacterial immunity.
